# Microbial mechanisms of carbon sequestration discrepancy between broadleaf and Moso bamboo forests

**DOI:** 10.3389/fmicb.2025.1580720

**Published:** 2025-06-03

**Authors:** Yaowen Xu, Jiejie Jiao

**Affiliations:** ^1^Zhejiang Academy of Forestry, Hangzhou, China; ^2^Zhejiang Hangzhou Urban Ecosystem Research Station, Hangzhou, China

**Keywords:** microbial-derived carbon, amino sugars, lignin phenols, mineral properties, soil organic carbon

## Abstract

In subtropical areas, broadleaf forests are being increasingly converted into Moso bamboo (*Phyllostachys pubescens*) forests. However, few studies have systematically compared soil organic carbon (SOC) between broadleaf and Moso bamboo forests. Therefore, we investigated SOC content and relative contributions of microbial and plant residues to SOC in broadleaf and Moso bamboo forests using biomarkers. The results show that the SOC content in Moso bamboo forest soil was 12.58% lower than that in adjacent broadleaf forest. Moreover, Moso bamboo forest soils also have less microbial-derived C but more plant-derived C compared with that in the broadleaf forest soil. The changes of microbial- and plant-derived C were mainly affected by soil properties. In particular, soil pH, ligninase/cellulase ratio, and mineral properties were the main factors regulating microbial-derived C, whereas mineral properties primarily controlled plant-derived C. Overall, our study reveals differences in C sequestration pathways between broadleaf and Moso bamboo forests, highlighting the potential to increase C storage through appropriate soil management, which provides a valuable reference for mitigating climate change.

## 1 Introduction

Soil organic carbon (SOC) constitutes a heterogeneous complex of biogeochemically active compounds generated by dynamic pedogenic processes. This carbon pool integrates both labile (e.g., microbial metabolites) and recalcitrant fractions (e.g., lignin derivatives) originating from plant residues, microbial biomass, and other biological inputs (Friedlingstein et al., 2020; Whalen et al., 2022). Persistent plant residues, such as lipids, have traditionally been regarded as notable sources of SOC owing to their chemical stability and resistance to microbial decomposition (Whalen et al., 2022). However, mounting evidence suggests that lignin phenols are only abundant in plant detritus and rarely accumulate in mineral soils (Liang et al., 2017; Wang et al., 2022). Furthermore, their low content in stable C pools with long turnover times (Sollins et al., 2009; Klotzbücher et al., 2014), indicating their labile nature. Over the past decade, conceptual models of SOC dynamics have transitioned from emphasizing selective preservation of recalcitrant plant-derived compounds (e.g., lignin and cutin) to prioritizing microbial-driven processes under the microbial carbon pump framework (Liang et al., 2017; Wang et al., 2022). In this paradigm, plant-derived inputs are converted by soil microbes into various microbial products, gaining recognition for microbial residues as precursors in the formation of SOC (Liu et al., 2021; Xu et al., 2023a; Hua et al., 2024). Therefore, identifying the distinct contributions of plant and microbial residues to SOC is essential for understanding the processes of SOC formation and stabilization that are heavily influenced by land use and environmental factors.

Global forest carbon storage reached 662 Gt, of which about 45% is stored in soil, such that even subtle changes in this vast C pool can have a substantial impact on the concentration of CO_2_ in the atmosphere, thereby affecting the global climate system (FAO, 2020). Land use change can profoundly affect soil organic carbon (SOC) storage, and it has caused CO_2_ emissions that are second only to the burning of fossil fuels, averaging 1.6 Gt y^−1^ over the past decade (2010–2019) (Friedlingstein et al., 2020). In China and other subtropical areas, broadleaf forests are important forest types with high biodiversity and C storage (Wang et al., 2007). In recent decades, however, vast areas of broadleaf forests have been converted into plantations, most notably Moso bamboo (*Phyllostachys pubescens*) forests (Xu et al., 2023a). In China, the cultivated area of bamboo spans 5.23 million ha, accounting for 69.78% of the country’s total bamboo forest area (Li et al., 2015). Owing to declining Moso bamboo prices and escalating labor expenses, numerous Moso bamboo plantations have been abandoned, increasingly posing a challenge for local authorities because of their minimal economic importance and aggressive spreading nature (Xu et al., 2023a). Broadleaf and Moso bamboo forests have distinct characteristics both above- and belowground, including plant biomass, litter quality, soil microbial communities, and soil physicochemical properties (Okutomi et al., 1996; Wang et al., 2016; Qin et al., 2017; Liu et al., 2022). These distinct characteristics will affect the relative contributions of microbial and plant-derived components to the organic carbon pool. However, it is still unclear how the transformation of broadleaf forests into Moso bamboo forests affects the roles of plant residue chemistry and microbial processes in controlling SOC dynamics (Liang et al., 2017).

To address this gap, we aimed to estimate the contributions of microbial and plant residues to SOC and compare the C sequestration pathways between the two forest types. In addition, plant biomass and soil microbial characteristics, mineral properties, and chemical properties were examined to identify the primary factors influencing microbial- and plant-derived C. Given that broadleaf forests have greater plant biomass than that of Moso bamboo forests, and the establishment of new forests often leads to the rapid loss of unstable C, we hypothesize that: (i) SOC content, plant residues, and microbial residues are higher in broadleaf forest than in Moso bamboo forest; (ii) microbial residues contribute more to SOC in Moso bamboo forest compared with broadleaf forest.

## 2 Materials and methods

### 2.1 Study area

The study site is located in Jiande City, Zhejiang Province, China (Supplementary Figure S1), at an elevation of 120–150 m. This region boasts a humid climate, characterized by a mean annual temperature of 16.7°C and an annual rainfall averaging 1,600 mm. The soil is categorized as red and yellow soil, with a thickness of 30–80 cm. Historically, this area was a broadleaf forest unmanaged by humans for approximately four decades and dominated by *Cyclobalanopsis glauca*, *Phoebe zhennan*, and *Castanopsis sclerophylla*, with an estimated tree density of 2,750 stems ha^−1^ (Supplementary Table S1). During the 1980s, this subtropical forest ecosystem underwent land-use conversion to Moso bamboo plantations, accompanied by sustained intensive management regimes. The anthropogenic practices included: (1) biennial application of NPK compound fertilizer (700–1,000 kg ha^−1^; N + P₂O_5_ + K₂O ≥45%), (2) deep tillage to 30–35 cm depth to disrupt subsurface soil layers, (3) annual harvesting of bamboo shoots during the sprouting season, (4) concurrent removal of understory vegetation and surface litter to minimize resource competition, and (5) selective harvesting of mature culms (>5 years old) to maintain stand productivity. Later, these Moso bamboo forests transitioned to unmanaged status. Over a minimum 15-year period, all anthropogenic management practices—including fertilization, tillage, selective harvesting, and litter removal—were completely ceased.

### 2.2 Experimental design and soil sampling

A paired-plot method was used to assess plant biomass in broadleaf and Moso bamboo forests. Each paired plot consisted of two adjacent 20 × 20 m plots: one in broadleaf forest and one in Moso bamboo forest, matched for similar topographical features (e.g., slope and elevation). In August 2022, 11 independent paired plots (totaling 22 plots, 11 per forest type) were established across a 60-ha area. To ensure spatial independence, paired plots were separated by at least 200 m from each other. The diameter at breast height and height of all trees in each plot were measured, and allometric equations were then used to calculate plant biomass for each plot (Qi et al., 2016).

Prior to soil sampling, litter above the soil surface was removed. To account for within-plot heterogeneity, five soil subsamples were collected from each plot (spaced 5 m apart in a grid pattern) at a depth of 0–15 cm using a 5 cm diameter soil drill. These subsamples were thoroughly mixed to form a single composite sample per plot. Consequently, the statistical analysis utilized 11 independent biological replicates per forest type (*n* = 11 for broadleaf forest; *n* = 11 for bamboo forest), corresponding directly to the 22 independent plots. After removing roots and stones, each soil sample was then divided into three separate sections: the first section was dried at 105°C until constant weight was achieved; second section was preserved at 4°C for analysis of phospholipid fatty acids (PLFAs) and enzyme activities; and third section was air-dried for 2 weeks before being milled to assess pertinent soil characteristics.

### 2.3 Soil physicochemical analyses

The SOC content was determined using the potassium dichromate heating oxidation method, whereas total phosphorus (TP) and total nitrogen (TN) contents were analyzed using an elemental analyzer (CHN-O-RAPID, Heraeus, Germany) (Xu et al., 2023a). Soil pH was measured using the potentiometric method (Li et al., 2018). The soil water content was determined using the oven-drying method. Dissolved organic carbon content was determined using 0.5 M K_2_SO_4_ extraction (Xu et al., 2023a). Soil cation exchange capacity was measured using ammonium salt exchange followed by hydrochloric acid titration. The clay content was analyzed using a laser diffraction particle size analyzer (MasterSizer 2000, Malvern Corporation, Malvern, United Kingdom). Iron and aluminum oxides in soil were extracted using a suitable solvent and identified using an inductively coupled plasma emission spectrometer (Optima 2000, PerkinElmer, Winchester, United Kingdom). Six types of iron and aluminum oxides were detected, including oxides with poor crystallinity (Fe_o_/Al_o_), free oxides (Fe_d_/Al_d_), and complexed oxides (Fe_p_/Al_p_).

### 2.4 C-degrading enzymes and soil microbial community

Assays were conducted in a 96-well microplate to measure the activities of four soil C-degrading extracellular enzymes categorized into two groups: ligninases serve as proxies for peroxidase and polyphenol oxidase activities, whereas cellulases are representative of the activities associated with β-1,4-glucosidases and β-1,4-D-cellobiohydrolases (Zhou et al., 2022).

Microbial PLFAs were extracted from approximately 8 g of air-dried soil by mixing it with a specific volume ratio of chloroform/methanol/phosphate buffer (1/2/0.8), adjusted to a pH of approximately 7.4. Phosphoric acid was added, followed by thorough shaking and incubation for 20 h. The methyl ester fatty acid samples were derived after a series of passivation and methylation steps, and analyzed using gas chromatography (Agilent 7890, Agilent Technologies, Wilmington, DE, United States) (Vestal and White, 1989). The concentration of PLFAs in each sample was calculated based on the 19/0 internal standard content, and the classification of different microbial communities is listed in Supplementary Table S2.

### 2.5 Determination of lignin phenols and amino sugars

The quantification of lignin-derived phenolic monomers was performed using an established alkaline CuO oxidation protocol optimized. Briefly, organic matter samples underwent oxidative degradation in 2 M NaOH with 0.5 g CuO at 155°C under nitrogen atmosphere for 3 h, effectively releasing lignin monomers while preserving aromatic structures. Reaction products were acidified to pH 1–2, extracted with ethyl acetate, and derivatized using bistrifluoroacetamide to enhance volatility for chromatographic analysis.

Lignin phenols were separated and quantified using an gas chromatography (Agilent 7890, Agilent Technologies) equipped with a flame ionization detector and a HP-5MS capillary column. The temperature program initiated at 80°C (1 min hold), ramped at 8°C min^−1^ to 250°C, then at 4°C min^−1^ to 300°C (10 min hold). Eight diagnostic phenolic monomers were identified based on retention times and co-injection with certified standards, categorized into three structural classes: vanillyl (V)-type monomers (vanillin, acetovanillone, and vanillic acid), syringyl (S)-type monomers (syringaldehyde, acetosyringone, and syringic acid), and cinnamyl (C)-type monomers (p-coumaric acid and ferulic acid). Quantification utilized response factors derived from external calibration curves with 3,5-dimethoxybenzoic acid as an internal standard. The following formula was used to calculate the amount of plant-derived C in the soil (Yang et al., 2022; Xu et al., 2023a):


(1) $$P=\frac{\frac{V-\text{type phenols}}{0.33}+\frac{S-\text{type phenols}}{0.9}+C-\text{type phenols}}{0.03\times \mathrm{SOC}}\times 100\%$$

V, S, and C denote carbon contents related to V-, S-, as well as C-type phenols (g kg^−1^); 3% denotes the minimal lignin content of the principal plant residues (Equation 1).

Amino sugars were determined via acid hydrolysis combined with gas chromatography (Zhang and Amelung, 1996). Briefly, 6 M HCl was added to freeze-dried soil (approximately 0.5 g) for hydrolysis and filtration. Myo-inositol (0.1 mL) was added. The pH value of the solution was adjusted to 6.7 and centrifuged at 2,500 r min^−1^ for 30 min to remove precipitation. The supernatant was freeze-dried (Labconco 6 L, Kansas City, MO, United States), and the remaining solid substance was dissolved in anhydrous methanol and centrifuged at 2,500 r min^−1^ for 30 min. The supernatant was transferred to a bottle, and the solution was dried with N_2_. The solution was then mixed with 1 mL deionized water and 0.1 mL N-methylglucamine, shaken, and freeze-dried again. Finally, amino sugar-related derivatives were determined via gas chromatography (Agilent 7890, Agilent Technologies).

Three amino sugars were identified to represent total amino sugars: glucosamine (GlcN), galactosamine (GaIN), and muramic acid (MurN) glucosamine. The fungal- and bacterial-derived C contents (g kg^−1^) were calculated using the following formulas (Liang et al., 2017):


(2) Fungal−derivedC=(GlcN/179.17−2×MurN/253.23)×179.17×9

(3) Bacterial−derivedC=MurN×45

Here, GlcN and MurN were measured in mg kg^−1^. The constants 179.17 and 253.23 mg kg^−1^ are the relative molecular weights of GleN and MurN, respectively. The conversion coefficients for GIcN to fungal-derived C and MurN to bacteria-derived C were 9 and 45, respectively. Microbial-derived C was calculated as the sum of fungal- and bacterial-derived C, as described in Equations 2, 3.

### 2.6 Statistical analyses

An independent sample t test was employed to ascertain differences in various components such as SOC, lignin phenols, amino sugars between broadleaf and Moso bamboo forests, whereas linear regression was employed to assess the correlations among (Ac/Al) v and (Ac/Al) s, (Ac/Al) v and total lignin phenols, and (Ac/Al) s and total lignin phenols. The relative importance of environmental variables in regulating microbial- and plant-derived C was determined using a random forest model. Finally, the regulatory pathways of these factors on microbial- and plant-derived C were determined using structural equation model (SEM). All statistical analyses were performed utilizing R 4.0.2.^1^

## 3 Results

### 3.1 Lignin phenol and amino sugar

**Figure 1:**
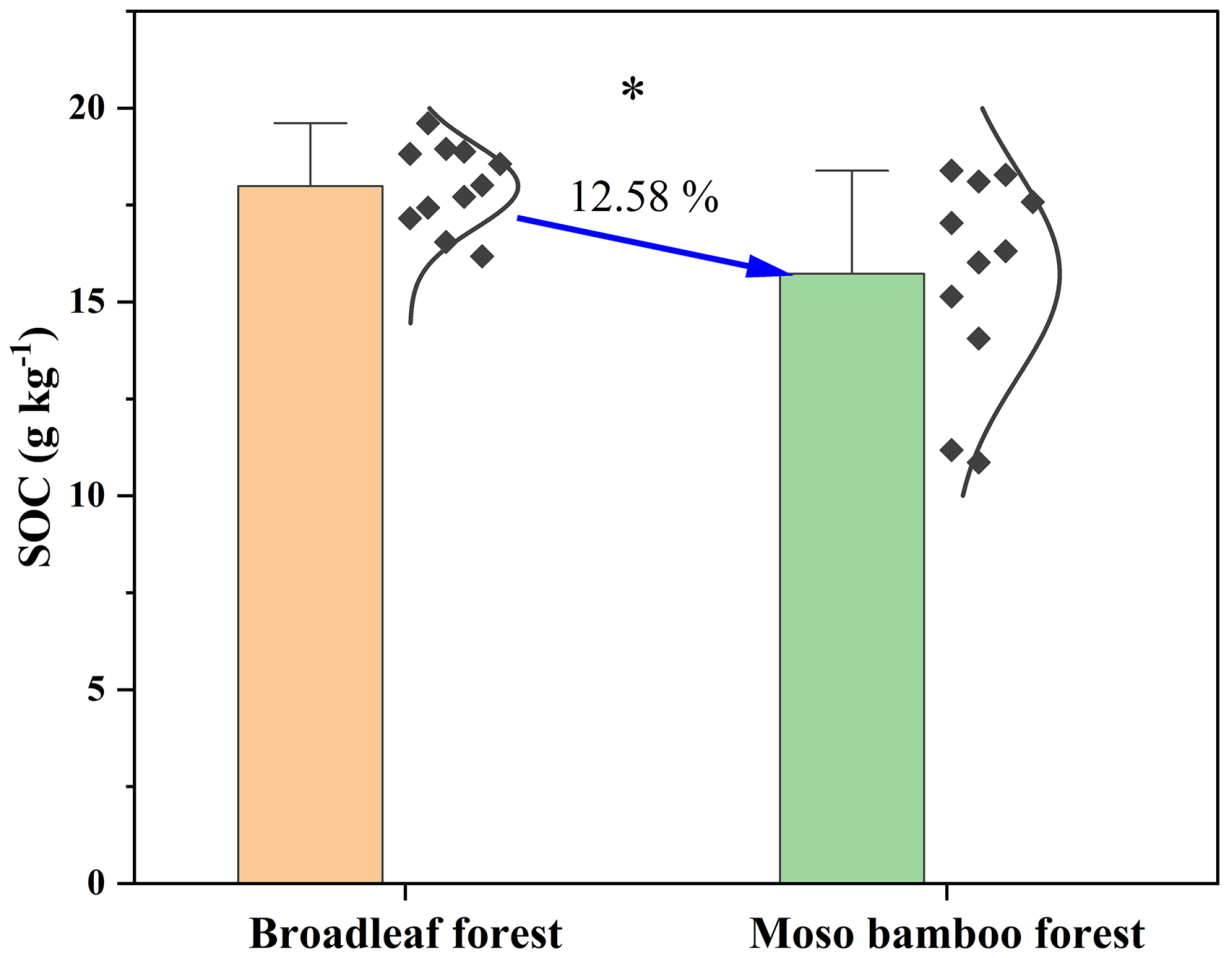
Contents of soil organic carbon in broadleaf and Moso bamboo forests. ^*^p < 0.05.

**Figure 2:**
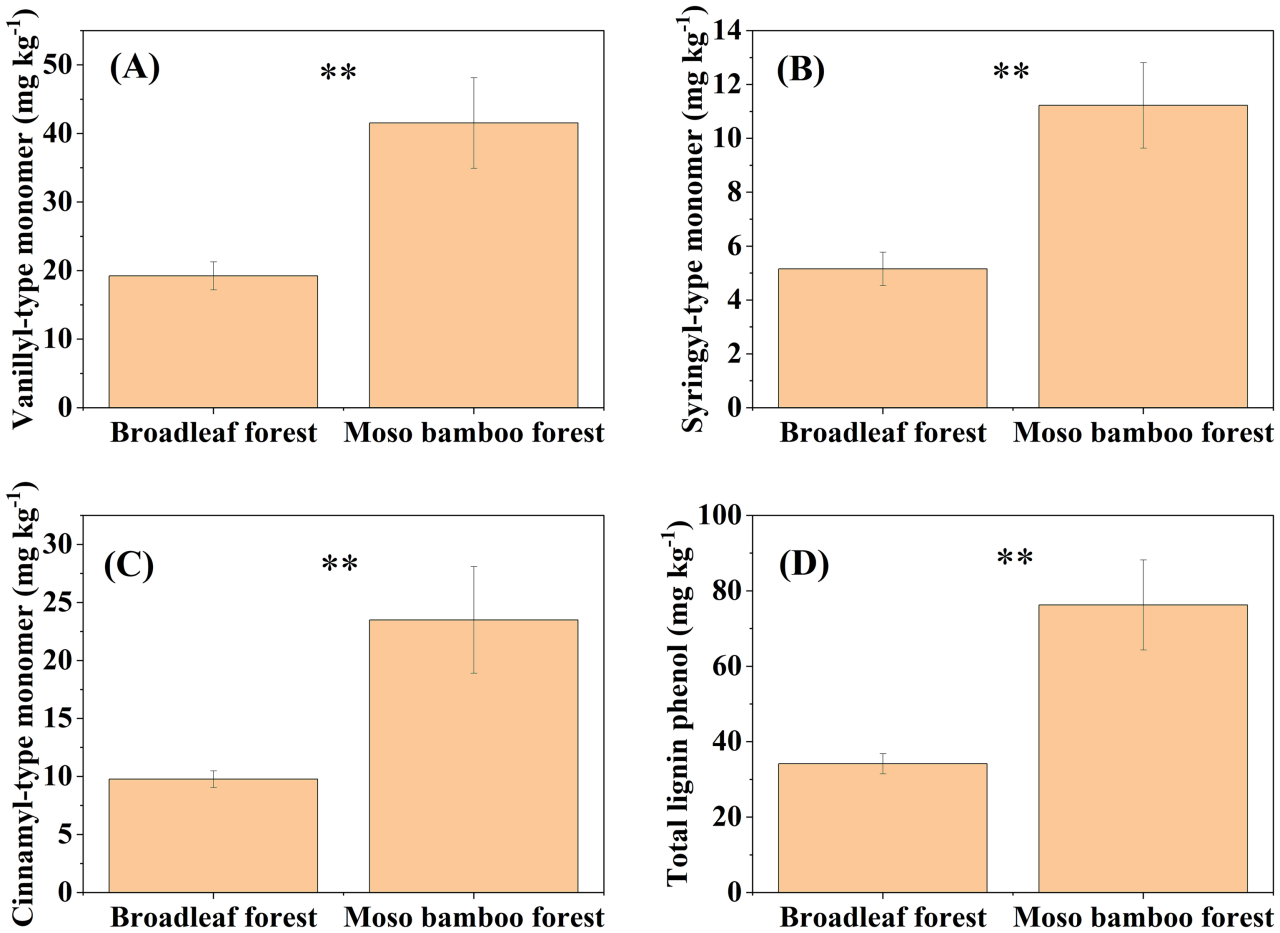
Contents of vanillyl-type monomer **(A)**, syringyl-type monomer **(B)**, cinnamyl-type monomer **(C)**, and total lignin phenols **(D)** in broadleaf and Moso bamboo forests. ^**^p < 0.01.

**Figure 3:**
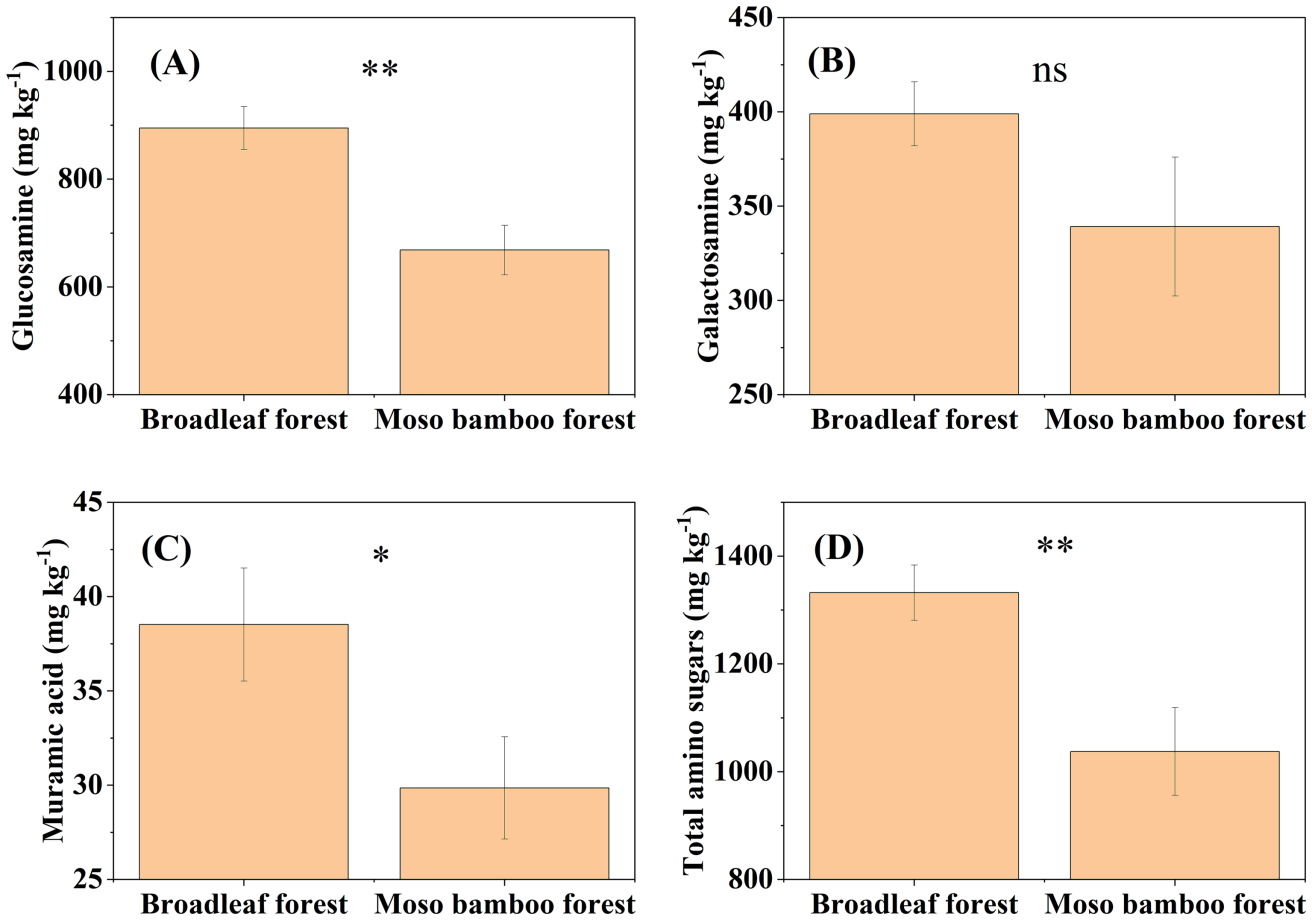
Contents of glucosamine **(A)**, galactosamine **(B)**, muramic acid **(C)**, and total amino sugars **(D)** in broadleaf and Moso bamboo forests. ^*^p < 0.05 and ^**^p < 0.01, ns represents not significant (*p* > 0.05).

**Figure 4:**
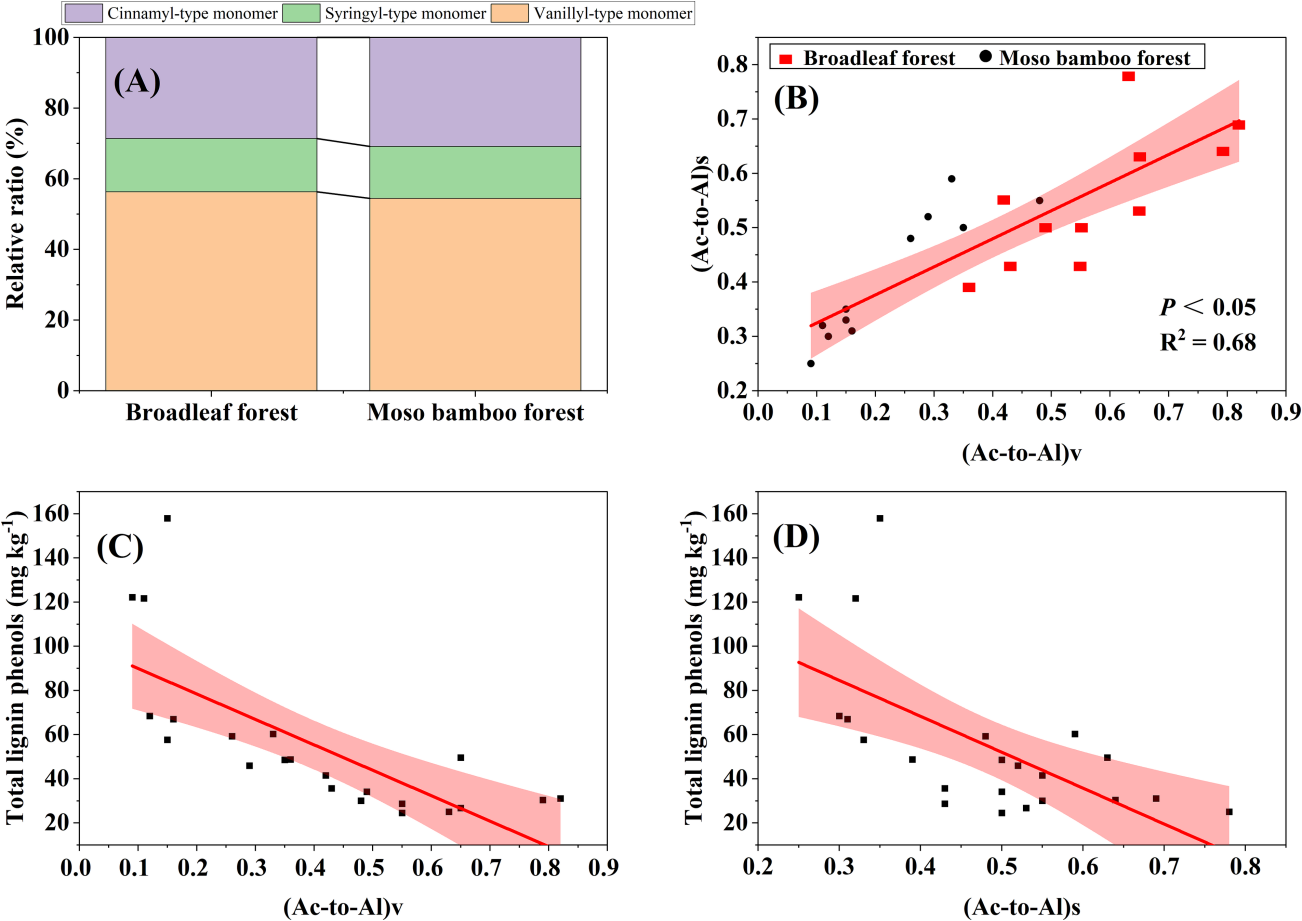
Proportion of the V-, S-, and C-type phenols in the total lignin phenols **(A)**, relationship between acid to aldehyde ratios of syringyl (Ac/Al)s and vanillyl (Ac/Al)v **(B)**, relationship between the total lignins and (Ac/Al)v **(C)**, and relationship between total lignins and (Ac/Al)s **(D)**. The red areas represent 95% confidence intervals, and the solid lines represent fitted regressions.

The SOC content in the broadleaf forest was 12.58% higher than that in the adjacent Moso bamboo forest (Figure 1). The lignin phenol contents (including V-, S-, and C-type and total lignin phenols) in the broadleaf forest were significantly lower than that in the Moso bamboo forest (*p* < 0.01; Figure 2). The total lignin phenol content in the Moso bamboo forest was more than twice that in the broadleaf forest (Figure 2D). The GlcN, MurA, and total amino sugar contents in the broadleaf forest were significantly higher than those in Moso bamboo forest (all *p* < 0.05; Figure 3). As shown in Figure 4A, the lignin phenol composition in both forests was dominated by V-type phenols (accounting for 54.46–56.30% of the total lignin phenols), followed by C- (28.61–30.82%), and S-type phenols (14.72–15.09%). The lignin phenols in broadleaf forest soil have higher (Ac/Al) v and (Ac/Al) s values than those in the Moso bamboo forest soil (Figure 4B). The (Ac/Al) v and (Ac/Al) s values were negatively correlated with the total lignin phenols (*p* < 0.05) across all plots (Figures 4C,D).

### 3.2 Contribution of microbial and plant residues to SOC

**Figure 5:**
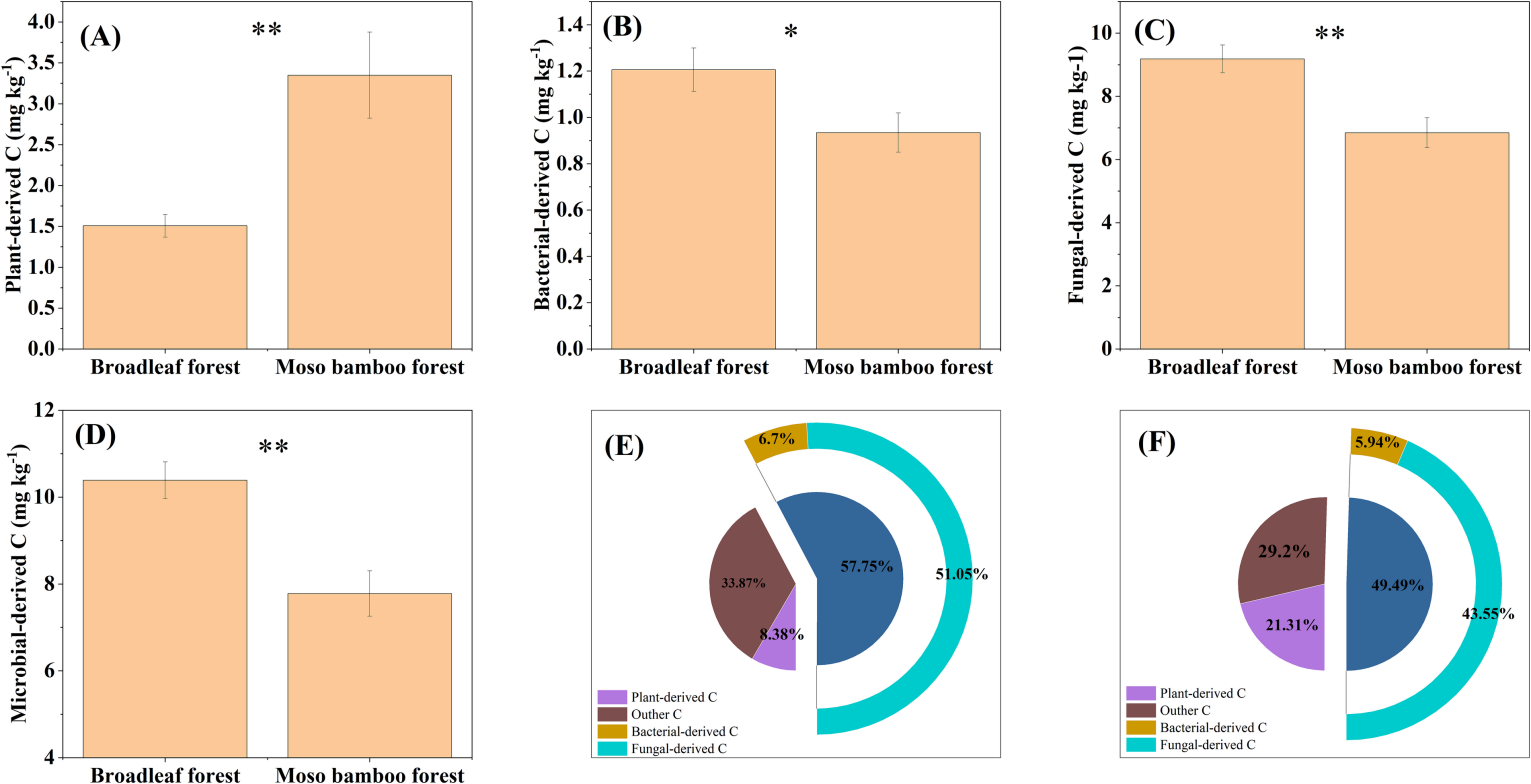
Contents of plant-derived carbon **(A)**, bacterial-derived carbon **(B)**, fungal-derived carbon **(C)**, and microbial-derived carbon **(D)** in broadleaf and Moso bamboo forests and the ratios of plant-, bacterial-, fungal-, and microbial-derived carbon to soil organic carbon in broadleaf **(E)** and Moso bamboo **(F)** forests. ^*^p < 0.05 and ^**^p < 0.01.

The plant-derived C content in broadleaf forest was significantly lower than that in the Moso bamboo forest (*p* < 0.01; Figure 5A). The bacterial-derived C content in broadleaf and Moso bamboo forest was 1.21 and 0.93 g kg^−1^, respectively (Figure 5B). The fungal-derived C content of the broadleaf and Moso bamboo forest was 9.18 and 6.85 g kg^−1^, respectively (Figure 5C). The fungal-, bacterial-, and microbial-derived C contents in the broadleaf forest were significantly higher than those in the Moso bamboo forest (all *p* < 0.05; Figures 5B–D). Comparative analysis revealed, the amount of plant-derived C in the Moso bamboo forest was 2.22 times that in the broadleaf forest, whereas the amount of microbial-derived C in the Moso bamboo forest was only 0.75 times that in the broadleaf forest (Figures 5A,D). Particularly, 29.2% of the SOC accumulated in Moso bamboo forest soil was plant-derived C, compared to just 8.38% in broadleaf forest soil (Figures 5E,F). Meanwhile, 57.75% of the SOC stored in the broadleaf forest was microbial-derived C, compared to just 49.49% in the Moso bamboo forest (Figures 5E,F).

### 3.3 Dominant determinants of microbial- and plant-derived C

**Figure 6:**
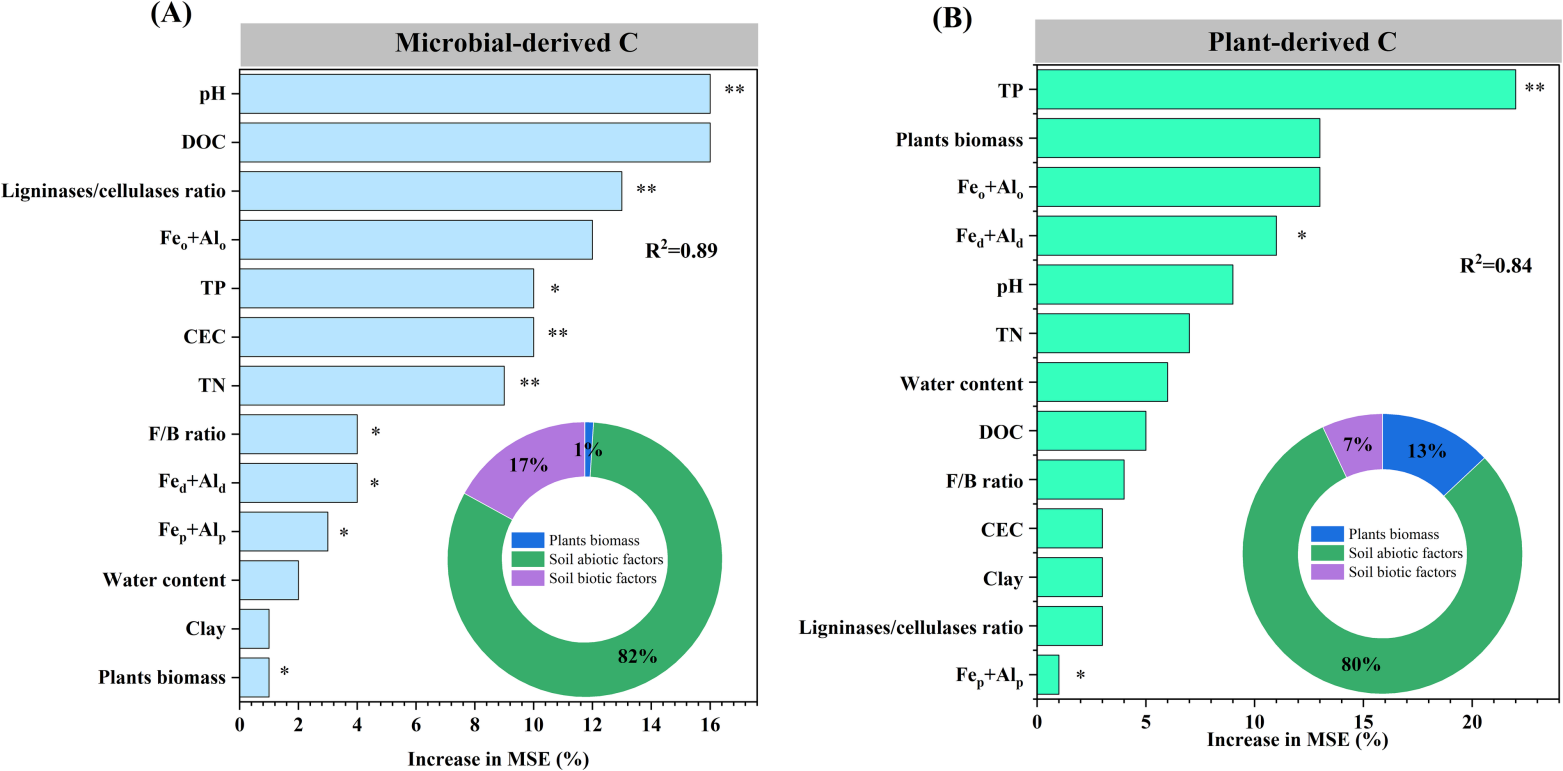
Random forest model was used to explore the environmental factors that drive changes in microbial- **(A)** and plant-derived carbon **(B)**. DOC, dissolved organic carbon; Fe_o_ + Al_o_, sum of poorly crystalline iron and aluminum oxides; TP, total phosphorus; TN, total nitrogen; CEC, cation exchange capacity; F, fungal; B, bacterial; Fe_d_ + Al_d_, sum of free iron and aluminum oxides; Fe_p_ + Al_p_, sum of complexed iron and aluminum oxides. ^*^p < 0.05 and ^**^p < 0.01.

The relative contributions of 13 variables reflecting the effects of plant biomass and soil abiotic and biotic factors on microbial- and plant-derived C were studied using a random forest model. Plant biomass and soil abiotic and biotic factors explained 1, 82, and 17% of the total variance in microbial-derived C and 13, 80, and 7% of the total variance in plant-derived C, respectively (Figures 6A,B). The most crucial factors explaining the contributions of microbial- and plant-derived C were pH and TP, respectively (*p* < 0.01; Figures 6A,B).

**Figure 7:**
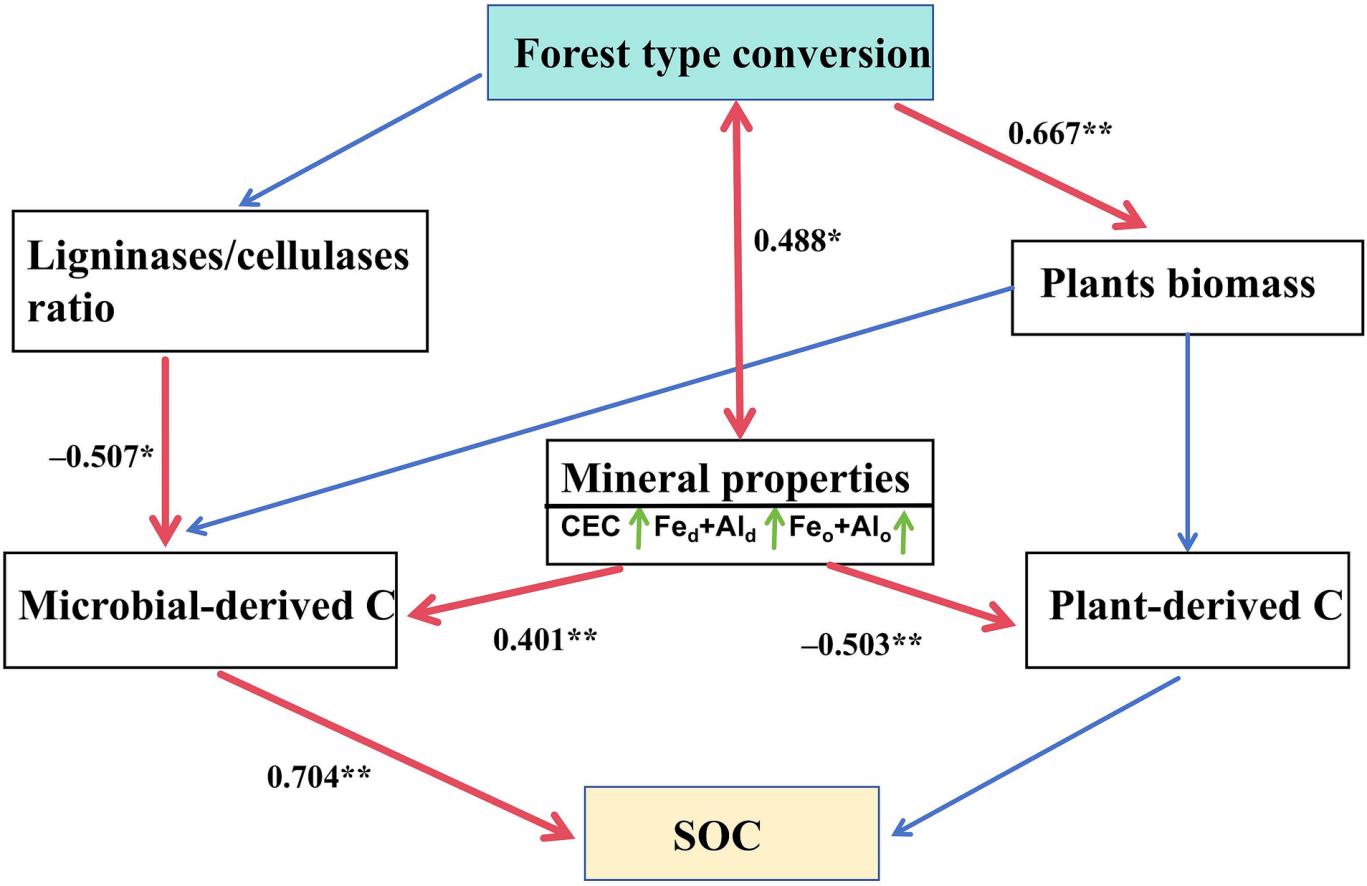
Structural equation model disentangling major pathways of environmental influences on plant- and microbial-derived carbon and soil organic carbon. Single-headed arrows indicate the hypothesized direction of causation. Red solid lines indicate significant relationships, whereas blue lines indicate non-significant relationships. Numbers are standardized path coefficients. Fe_o_ + Al_o_, sum of poorly crystalline iron and aluminum oxides; CEC, cation exchange capacity; Fe_d_ + Al_d_, sum of free iron and aluminum oxides. ^*^p < 0.05 and ^**^p < 0.01. df = 11, *χ*^2^/df = 1.095, *p* = 0.36, RMSEA = 0.067, CFI = 0.981.

SEM was used to test hypothesized direct/indirect effects of soil properties and enzyme activities on C sources. For microbial-derived C, ligninase/cellulase ratio and mineral properties had direct and positive effects. For plant-derived C, mineral properties had direct and negative effects. The change in SOC was primarily driven by a change in microbial-derived C (Figure 7).

## 4 Discussion

### 4.1 Comparison of SOC between broadleaf and Moso bamboo forests

Consistent with our first hypothesis, higher SOC content in the broadleaf forest than that in the Moso bamboo forest. Several factors attributed to the intensive management practices may explain the reduced SOC content in Moso bamboo forest. First, anecdotal evidence from local farmers suggests that tillage to a depth of approximately 30 cm leads to considerable soil disturbance. This disturbance accelerates SOC decomposition by undermining physical protection, increasing contact with air, and increasing the intensity of erosion after rainfall (Guan et al., 2015). Second, fertilization practices in bamboo management can accelerate the mineralization of SOC and leaching of soluble organic matter, thereby hindering the accumulation of SOC (Shao et al., 2023). Third, the reduction in litterfall, caused by high-frequency thinning and selective cutting during the management of Moso bamboo forest, results in decreased in soil C input.

### 4.2 Plant- and microbial-derived C

We found that SOC of the Moso bamboo forest contained more plant-derived C and less microbial-derived C compared with that of the broadleaf forest. Variations in SOC sourcing could be attributed to differing tree species compositions between the two forest types. Several studies suggest that expansion of bamboo forests reduces species richness and litter production, leading to reduced substrate availability in the soil matrix. The decreased substrate accessibility limits microbial turnover and delays microbial residue production while simultaneously promoting the accumulation of plant residues (Zhao et al., 2021). In addition, microorganisms preferentially utilize small and unstable organic molecules as C sources, which are considerably present in the litter of broadleaf forests (Li et al., 2020). Contrary to the second hypothesis, microbial residues contribute less to SOC in Moso bamboo forest compared with broadleaf forest (Figures 5E,F). This may reflect two management-induced constraints: (1) tillage (30–35 cm depth) disrupts fungal hyphal networks and reduces mycorrhizal colonization rates, potentially decoupling plant-microbe C allocation (Xu et al., 2023a). (2) Compound NPK fertilization in bamboo plantations likely shifted microbial communities toward fast-growing r-strategists, prioritizing labile C mineralization over stable microbial necromass accrual (Xu et al., 2023b).

An analysis of global forests reveals that microorganisms contribute 35% of the SOC (Wang et al., 2021). In this study, microbial-derived C accounted for 49.49–57.75% of the SOC, whereas plant-derived C ranged from 8.38–21.31%. Consequently, the enhanced capacity of broadleaf forest to sequester SOC, in comparison to adjacent Moso bamboo forest, is predominantly due to the preferential accumulation of microbial-derived C. This is evident from the fact that the total amino sugars content in broadleaf forests is 28.43% greater than in Moso bamboo forest.

In this study, the contents of fungal-derived C in both broadleaf and Moso bamboo forests was higher than that from bacterial-derived C, and a similar phenomenon has been observed in in analyses of forests on a global scale (Wang et al., 2021). Several factors may explain this discrepancy: (i) fungal residues can become stabilized in the soil by forming fungal necromass–tannin complexes (Xu et al., 2023a), (ii) fungal cell wall fragments can efficiently form aggregates of large molecules with smaller surface area-to-volume ratio, which are more resistant to decomposition than bacteria (Vidal et al., 2021), and (iii) the low pH value of forest soil may stimulate fungal turnover and thus increase the production of fungal residues (Hu et al., 2020). Furthermore, PLFAs exhibited dominance in broadleaf forest soils (Supplementary Table S2), correlating with their functional specialization in decomposing recalcitrant plant-derived polymers (e.g., lignin and cellulose) and facilitating necromass stabilization through biochemical protection mechanisms (Hu et al., 2020). This aligns with established fungal traits, including the production of oxidative enzymes for lignocellulose breakdown and the synthesis of hydrophobic cell wall components that enhance necromass persistence in soil organic matter pools. In contrast, bacterial communities exhibited a metabolic preference for labile substrates (e.g., root exudates and dissolved organic carbon), thereby accelerating carbon turnover rates and reducing stabilization efficiency (Vidal et al., 2021).

### 4.3 Microbial- and plant-derived C kinetics

Microbial- and plant-derived C were primarily explained by abiotic soil factors. In particular, soil pH, mineral properties, and ligninase/cellulase ratio were main drivers of microbial-derived C. It is now well established that the planting Moso bamboo increases soil pH value (Ouyang et al., 2022). Contrastingly, broadleaf forest has lower soil pH, which provide a suitable environment to produce root exudates, and favors the microbial-derived C production (Hu et al., 2020). Furthermore, lower soil pH enhances microbial C use efficiency, thereby promoting the production of microbial residues (Jones et al., 2019). Mineral properties (mainly Fe/Al oxides) facilitate the accumulation of microbial-derived C in three ways: first, Fe/Al oxides provide microbes with protection, survival of substrates, and electrons necessary for microbial growth, thus promoting microbial turnover (Uroz et al., 2015); second, the binding sites on the mineral surface can inhibit the ability of enzymes to degrade and directly reduces the breakdown of organic matter (Zimmerman et al., 2004); third, Fe/Al oxides typically form a stable complexes with organic matter, which are less prone to oxidative degradation and biodegradation. Along with soil pH and mineral properties, the role of C-degrading enzyme activity in regulating spatial changes in microbial-derived C should not also be overlooked (Xu et al., 2023b). SEM showed that the reduction of ligninase/cellulase ratio promoted the preservation of microbial-derived C (Figure 7). The change in the ligninase/cellulase ratio reflects the change in microbial survival strategies because ligninase synthesis is more energy-intensive than cellulase synthesis (Chen et al., 2020). Therefore, a decrease in ligninase/cellulase ratio suggests that microorganisms are reducing their investment in resource acquisition, allotting more resources to growth, thereby promoting faster formation and accumulation of microbial-derived C (Chen et al., 2020). Overall, these results highlight the importance of soil pH, mineral characteristics, and ligninase/cellulase ratio in shaping the production and accumulation of microbial-derived C.

The higher (Ac/Al)s and (Ac/Al)v ratios in the broadleaf forest reflect a higher degree of lignin phenols oxidation, suggesting that plant-derived C decomposes more rapidly in broadleaf forest soil than in Moso bamboo forest soil (Angst et al., 2021). Lignin, mainly found in particulate organic matter that lacks mineral properties, may be released through reduction and dissolution of metallic oxides (Dai et al., 2022). Consequently, plant-derived C is negatively associated with mineral properties, supporting our findings (Figure 7). Therefore, SOC accumulation in Moso bamboo forests may be more unstable and susceptible to perturbations than in broadleaf forests (Shao et al., 2023). Our finding highlights the need for developing effective management strategies to prevent large C losses in Moso bamboo forests, particularly those associated with plant-derived C.

### 4.4 Limitations

Although this study revealed the key differences in carbon sequestration pathways between broad-leaved forests and bamboo forests, several limitations warrant attention. First, while we observed management-driven shifts in microbial community structure, the underlying functional gene expression mechanisms remain unresolved. Future studies employing metatranscriptomics or single-cell RNA sequencing could elucidate changes in microbial metabolic priorities. Second, although our sampling focused on the topsoil (0–15 cm)—the horizon with maximal plant litter inputs and microbial activity—this design may underestimate subsoil (>20 cm) contributions in deep-rooted systems. Future studies should integrate stratified soil profiling to resolve depth-dependent SOC mechanisms. Third, while the paired-plot design (*n* = 11 pairs) minimized environmental heterogeneity confounders, limited replication and potential spatial autocorrelation among adjacent plots may constrain the generalizability of our findings. Validating these patterns across broader geographic gradients with larger sample sizes (*n* > 30) would help disentangle scale-dependent drivers of SOC dynamics.

## 5 Conclusion

Our study reveals critical differences in SOC sequestration pathways between broadleaf and Moso bamboo forests, with microbial-derived C playing a central role in shaping SOC dynamics. We demonstrate that the lower SOC content in Moso bamboo forests compared to broadleaf forests is primarily driven by a substantial reduction in microbial-derived C, underscoring the pivotal influence of microbial activity on SOC accumulation. This decline likely stems from altered microbial community composition, reduced microbial C production, or shifts in microbial metabolic efficiency under Moso bamboo-dominated ecosystems. Restoring no-till practices in Moso bamboo forests may mitigate microbial C loss by reducing soil disturbance. Our findings also advance the understanding of subtropical forest C sequestration potential, stressing the need to integrate microbial biomarkers and activity metrics into predictive models to refine climate mitigation strategies. Future research should explore microbial functional traits and their responses to land-use change to unlock targeted interventions for SOC restoration.

## Data Availability

The original contributions presented in the study are included in the article/Supplementary material, further inquiries can be directed to the corresponding authors.

## Footnotes

^1^ https://www.r-project.org/
