# Supplementary material for: Microbial mechanisms of carbon sequestration discrepancy between broadleaf and Moso bamboo forests
Source: Front Microbiol. 2025 Jun 3;16:1580720. doi: 10.3389/fmicb.2025.1580720 (PMC12170563; doi:10.3389/fmicb.2025.1580720)
Supplement: Supplementary file 1 [file Data_Sheet_1.docx]

Supplementary Material

# Supplementary Figures and Tables

## Supplementary Figures

**
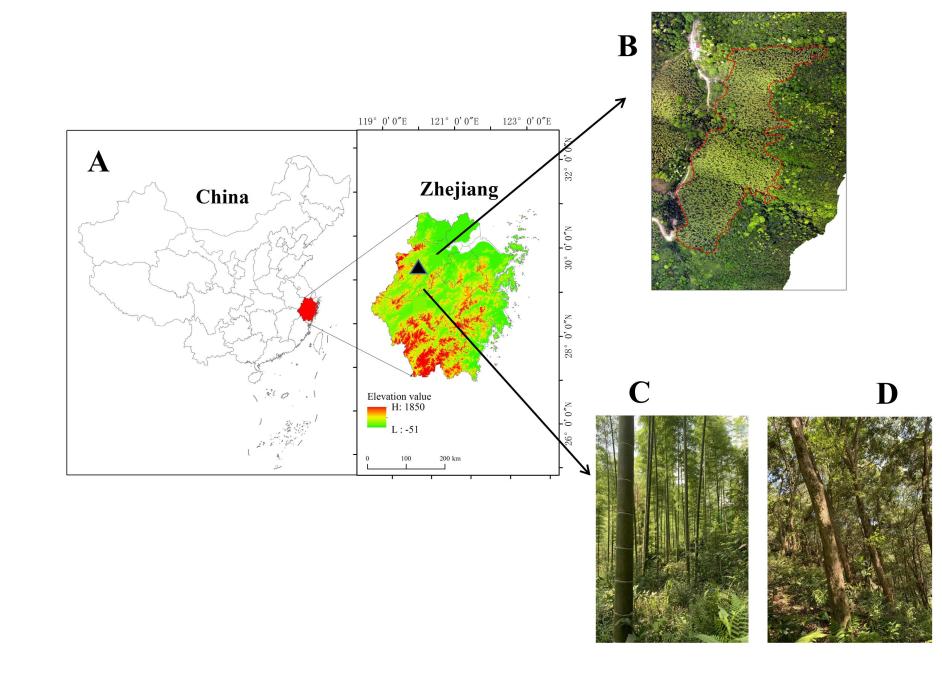
**

**Supplementary Figure 1.** The study area is located in the Jiangde, Zhejiang Province, China (A), top view of the study site (B), Moso bamboo forest (C), broadleaf foresty (D).

## Supplementary **Table**

**Supplementary Table 1.** Characteristics of sample plots

| Type of sample plot | Number | Average altitude (m) | Number of living tree | Major understory vegetation |
| --- | --- | --- | --- | --- |
| Moso bamboo forest | 11 | 123.67 | 3015 | *Viola verecunda, Paederia scandens, Arthraxon hispidus* |
| Broadleaf forest | 11 | 124.13 | 2750 | *Paederia scandens, Rhododendron simsii, Osmunda japonica, Camellia japonica,* |

**Supplementary Table 2.** The phospholipid fatty acids (PLFAs) detected in this study

| Category | PLFA biomarkers |
| --- | --- |
| Bacteria | 14:00, 15:00, 16:00, 17:00, 18:00, i19:0, c19:0ω8c,10Me19:1ω7c |
| Fungi | 18:1ω9c, 18:2ω6c, 18:3ω3c |

**Supplementary Table 3.** Environmental factors characteristics

| Item | Broadleaf forest | Moso bamboo forest |
| --- | --- | --- |
| pH value | 4.64±0.06 b | 5.05±0.08 a |
| TN (g kg^–1^) | 1.57±0.04 a | 1.42±0.1 a |
| SOC (g kg^–1^) | 17.99±0.33 a | 15.72±0.81 b |
| TP (g kg^–1^) | 0.37±0.01 a | 0.28±0.01 b |
| DOC (mg kg^–1^) | 206.11±10.09 a | 194.80±8.63 a |
| CEC (cmol kg^–1^) | 15.83±0.59 a | 13.97±0.62 b |
| Clay (%) | 32.65±0.94 a | 29.46±0.44 b |
| Ligninase (μmol h^–1^ g^–1^) | 2.48±0.25 b | 3.27±0.12 a |
| Cellulase (μmol h^–1^ g^–1^) | 0.52±0.04 a | 0.5±0.04 a |
| Ligninase/Cellulase ratio | 5.38±0.88 a | 6.58±0.5 a |
| Fe_d_+Al_d_ (g kg^–1^) | 4.31±0.17 a | 3.78±0.27 a |
| Fe_p_+Al_p_ (g kg^–1^) | 5.04±0.46 a | 3.87±0.15 b |
| Fe_o_+Al_o_ (g kg^–1^) | 4.28±0.14 a | 3.72±0.17 b |
| Plants biomass (t ha^–1^) | 130.06±7.75 a | 77.74±11.99 b |
| B (nmol g^–1^) | 4.31±0.54 b | 5.89±0.43 a |
| F (mg kg^–1^) | 1.04±0.13 b | 1.49±0.06 a |
| F/B ratio | 0.24±0.01 a | 0.26±0.01 a |

Note: The values are means ± SE. Different lowercase letters indicate significant differences (*P* < 0.05). TN: total nitrogen, TP: total phosphorus, DOC: dissolved organic carbon, CEC: cation exchange capacity, SOC: soil organic carbon, Fe_d_+Al_d_: sum of free iron and aluminum oxides, Fe_p_+Al_p_: sum of complexed iron and aluminum oxides, Fe_o_+Al_o_: sum of poorly crystalline iron and aluminum oxides, F: fungal, B: bacterial.
